# Bile Acid Metabolism Regulates Ovarian Function: Networks and Reproductive Health Applications

**DOI:** 10.1017/erm.2026.10035

**Published:** 2026-02-09

**Authors:** Wei Liu, Bo Zhang

**Affiliations:** Reproductive Medicine Center, Tongji Hospital, Tongji Medical College, Huazhong University of Science and Technology, Wuhan, China

**Keywords:** bile acids, endoplasmic reticulum stress, gut–BAs–ovary axis, ovarian function, polycystic ovary syndrome

## Abstract

**Background:**

Bile acids (BAs) are crucial metabolic regulators and signaling molecules involved in lipid metabolism and ovarian function. They primarily affect follicular development, steroidogenesis, and oocyte maturation through systemic circulation and transporter-mediated uptake (e.g., NTCP, ASBT) rather than local ovarian synthesis. Increasing evidence indicates that BA dysregulation is associated with multiple reproductive pathologies.

**Methods:**

This review is based on the authors’ own work and a comprehensive PubMed search of the literature to date on bile acids and female reproduction. PubMed was searched using the terms “bile acid AND ovary,” “bile acid AND oocyte,” and “bile acid AND reproduction.” Retrieved records were screened for relevance to ovarian physiology and pathology, including folliculogenesis, steroidogenesis, granulosa cell function, oocyte maturation, and reproductive disorders, and 51 articles were ultimately included in this review.

**Results:**

Studies show significant BA dysregulation in reproductive disorders. In polycystic ovary syndrome (PCOS), elevated glycochenodeoxycholic acid (GCDCA) and taurocholic acid (TCA) correlate with hyperandrogenemia. Excessive BAs can induce endoplasmic reticulum (ER) stress and granulosa cell apoptosis; for example, glycodeoxycholic acid (GDCA) promotes a BAX/BCL-2 imbalance and may accelerate follicular atresia. In contrast, protective BAs such as ursodeoxycholic acid (UDCA) and tauroursodeoxycholic acid (TUDCA) alleviate ER stress and oxidative damage and may improve oocyte quality. Mechanistically, BAs regulate steroidogenic enzymes (e.g., StAR, CYP11A1) via the nuclear receptor FXR and modulate ovarian function through pathways including EGF–ERK1/2 and PERK–ATF4. Moreover, the gut–BA–ovary axis has emerged as a metabolic hub linking environmental factors to reproductive function, potentially contributing to PCOS pathogenesis and ovarian reserve decline through an integrated regulatory network.

**Conclusions:**

BA-mediated signaling networks play important roles in ovarian physiology and reproductive disease. BAs and BA-related pathways may serve as novel biomarkers and therapeutic targets for reproductive disorders.

## Introduction

BAs are a structurally diverse group of amphipathic molecules, with over 60 distinct species identified in mammals to date. Their primary function is to facilitate the digestion and absorption of dietary lipids and fat-soluble vitamins in the small intestine. Synthesized in the liver and stored in the gallbladder, BAs are released into the intestinal lumen upon food intake. While most undergo enterohepatic re-circulation for re-use, a small fraction is excreted as waste (Ref. 1). Traditionally viewed as mere digestive agents, emerging research has revealed their critical role as signalling molecules that mediate finely tuned inter-organ communication – from the liver (their site of synthesis) to the gut (where they are modified by microbiota) and then via the circulatory system to nearly all organs, where they exert pleiotropic physiological effects (Ref. 2).

The structural diversity of BAs not only reflects the complexity of their synthesis and metabolism (e.g., classic vs. alternative pathways, microbial modification), but also suggests that distinct BA species may possess unique bioactivities. For instance, specific BAs can modulate metabolism, immunity and inflammatory responses by activating nuclear receptors (e.g., FXR) or membrane receptors (e.g., TGR5) (Ref. 2). These multifaceted properties underlie their significant alterations – and even pivotal roles – in various diseases, including metabolic disorders (Ref. 3), neurodegenerative diseases (Ref. 1), kidney diseases (Ref. 4) and inflammatory bowel disease (Ref. 5), positioning them as potential diagnostic markers or therapeutic targets (Ref. 6).

Notably, the regulatory effects of BAs on the reproductive system are increasingly recognized, particularly their roles in the follicular microenvironment. Recent studies have shown that follicular fluid (FF) contains BA concentrations higher than those in serum, with a predominance of primary BAs, indicating that BA homeostasis within the follicle is not solely determined by passive diffusion (Ref. 7). These observations raise important questions regarding the origin and regulatory mechanisms of BAs in the ovarian follicular environment. This phenomenon may influence follicular development, oocyte maturation and luteal function, closely linking BAs to female reproductive health. This review summarizes the sources, regulatory mechanisms and potential roles of BAs in FF, offering novel perspectives for research on reproductive physiology and related disorders.

## Sources of BAs in the ovary

A ground-breaking study in 2009 first proposed that human ovarian follicles might possess the capacity for local BA synthesis. The study reported the presence of key enzymes in the classical pathway (CYP7A1) and alternative pathway (CYP27A1/CYP7B1) in granulosa cells, along with detectable BA production in vitro under cholesterol-stimulated conditions, suggesting potential competition with steroidogenesis for cholesterol substrates (Ref. 7). However, subsequent rigorous experimental validation published in 2019 has revealed limitations in this hypothesis. It was demonstrated that ovarian granulosa cells notably lack functional *CYP7A1* expression and exhibit minimal alternative pathway enzyme activity, with no detectable BA production in serum-free culture systems (Ref. 8). Consistent with the absence of functional intra-ovarian BA synthesis, accumulating evidence indicates that BAs detected in FF predominantly originate from systemic circulation. Specifically, BA concentrations in FF show a significant correlation with serum levels (*r*_s_ = 0.186), and multiple BA transporters, including NTCP, ASBT and ABCC3, are selectively expressed in ovarian tissues. These findings support a transporter-mediated uptake mechanism, whereby circulating BAs are actively imported into the follicular microenvironment. Despite the suppression of local synthesis, primary BAs are enriched approximately twofold in FF compared with serum, further suggesting selective accumulation rather than passive diffusion alone.

**Figure 1.**
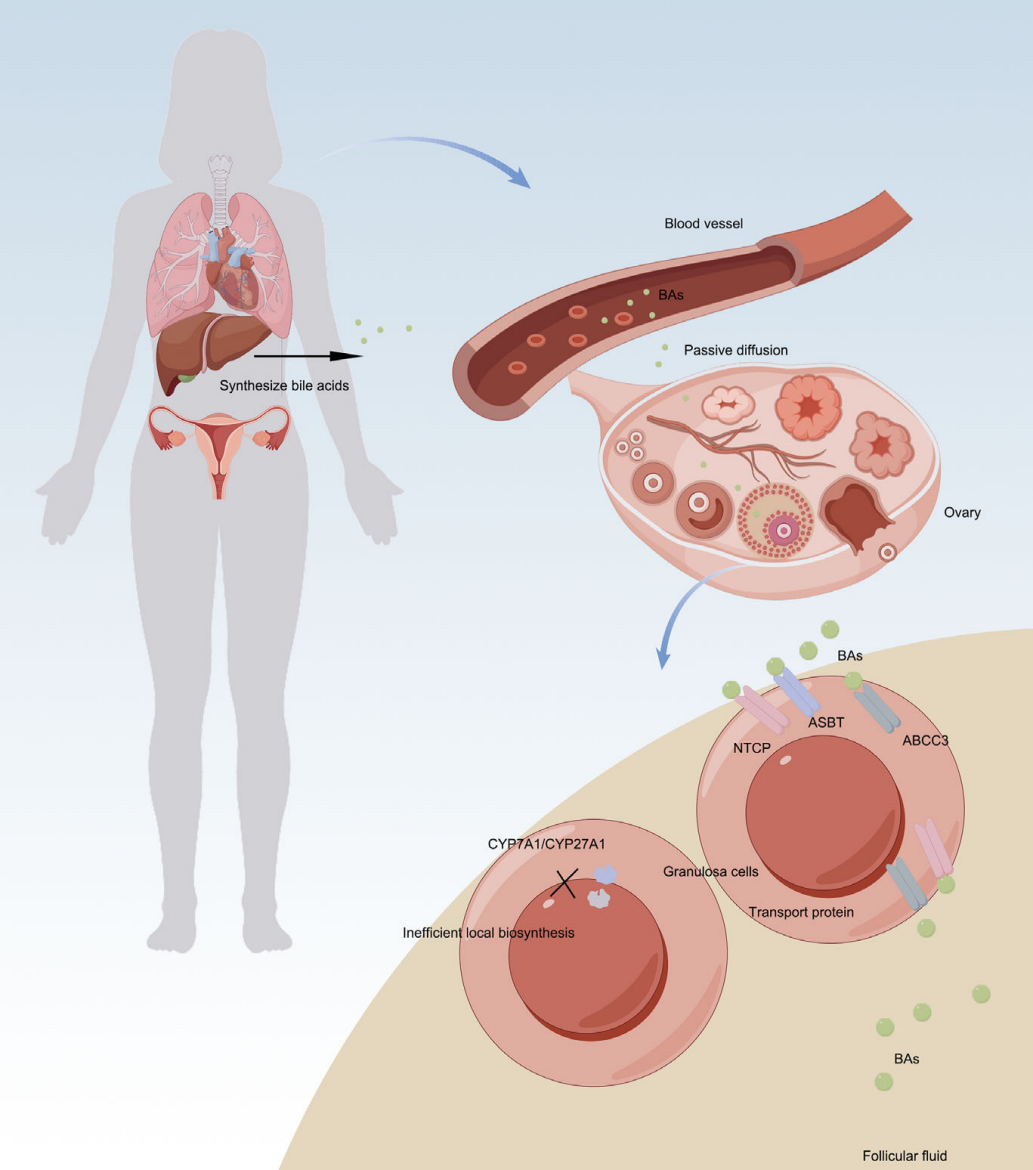
Sources and regulatory mechanisms of BAs in ovary. **A.** Recent evidence has revised the conventional view of ovarian BA synthesis: Local production is minimal – granulosa cells lack functional CYP7A1 expression and BA generation is negligible in serum-free culture systems. **B.** Ovarian BAs are primarily derived from systemic circulation via: a. passive diffusion; b. active transport mediated by NTCP/ASBT/ABCC3 transporters. This paradigm shift from ‘de novo synthesis’ to ‘transporter-mediated uptake’ establishes BAs as endocrine regulatory factors in follicular physiological activities.

Collectively, these observations indicate a conceptual shift from an initial ‘intraovarian synthesis’ hypothesis to a ‘transporter-mediated uptake’ model as the predominant source of follicular BAs, highlighting the physiological significance of BAs as endocrine regulators within the ovarian follicular microenvironment (Figure 1).

## BA metabolism dysregulation impairs ovarian function: multi-species evidence from follicular microenvironment imbalance to steroidogenic disruption

Fatty acid-binding protein 6 (Fabp6), conventionally known as a BA-binding protein in the distal small intestine that plays a crucial role in maintaining BA homeostasis, is also expressed in ovarian granulosa and luteal cells. Gonadotropins have been shown to up-regulate *Fabp6* expression in antral follicles. Studies using knockout mice demonstrate that *Fabp6* deficiency significantly impairs superovulation responses, highlighting its crucial role in ovulation and fertility while simultaneously underscoring the importance of BA homeostasis for ovarian function (Ref. 9). Regarding high-fat, high-sugar (HFHS) diet-induced ovarian dysfunction in mice, research revealed that BA metabolism and other critical pathways, including steroidogenesis, exhibit a biphasic response – characterized by initial up-regulation in the short term (1–4 weeks) followed by persistent suppression upon prolonged HFHS feeding (8–16 weeks). These changes closely correlate with the progressive deterioration of oocyte mitochondrial function and quality, thereby directly highlighting the essential role of BA metabolism in maintaining ovarian follicular homeostasis (Ref. 10). Moving to human studies, a metabolomic study involving women with latent genital tuberculosis (LGTB) demonstrated that LGTB impairs ovarian reserve function by disrupting BA metabolic pathways. Detailed analysis revealed significant alterations in primary BA biosynthesis within follicular fluid of LGTB patients. This metabolic disturbance was clinically correlated with diminished ovarian reserve markers (including reduced AMH levels and fewer antral follicles) and aberrant ovarian stimulation responses (manifesting as elevated gonadotropin requirements). Compared to healthy controls, LGTB-specific metabolic re-modelling suggests that dysregulated BA metabolism acts as a pivotal molecular hub, potentially compromising ovarian function through interference with steroid hormone biosynthesis and other reproduction-critical metabolic networks (Ref. 11). Animal experiments have revealed that significantly elevated BA levels in the dominant follicles of lactating dairy cows, accompanied by up-regulated expression of BA transporter *SLC10A2* and receptor *GPBAR1*. This abnormal activation of the BA signalling pathway, when synergizing with reduced IGF-1 signalling and metabolic stress, may impair ovarian function by disrupting the follicular microenvironment. These findings elucidate one of the key mechanisms underlying fertility decline in lactating cows (Ref. 12). At the molecular level, a fundamental study revealed that BAs (e.g., cholic acid, CA) significantly impair ovarian function by activating the farnesoid X receptor (FXR) signalling pathway. In a mouse model, CA treatment reduced ovarian weight, disrupted the oestrous cycle and decreased antral follicle and corpus luteum counts, all of which were accompanied by diminished progesterone and oestradiol levels. Mechanistically, CA up-regulated ovarian *FXR* expression while suppressing key steroidogenic genes (*StAR*, *CYP11A1* and *HSD3B1*). These findings demonstrate that BA dysregulation disrupts steroidogenesis and follicular development via FXR signalling, thereby identifying it as a critical pathological mechanism in ovarian dysfunction (Ref. 13).

## BA metabolism and follicular atresia

Recent studies have established BAs as critical metabolic regulators in ovarian physiology. This understanding originated from the seminal discovery that BAs transcriptionally suppress the cholesterol-metabolizing enzyme CYP7A1 through FXR receptor activation (IC50 = 10–25 μM) (Ref. 14). Subsequent investigations identified a distinctive BA profile in rat ovaries dominated by non-amidated lithocholic acid (LCA) and β-muricholic acid (β-MCA), showing a composition markedly different from hepatic–enterohepatic circulation patterns (Ref. 15). Remarkably, this ovary-specific profile dominated by unconjugated BAs suggests the potential existence of FXR-independent mechanisms regulating local cholesterol metabolism and steroidogenesis in follicular microenvironments. Further research has revealed a clear association between BA dysregulation and follicular atresia. Bile duct ligation (BDL) animal models demonstrated characteristic pathological alterations in reproductive organs: (1) suppressed testosterone synthesis; (2) elevated oxidative stress markers (reactive oxygen species, lipid peroxides and protein carbonyls) accompanied by reduced glutathione and (3) mitochondrial dysfunction (depolarization and decreased ATP production) (Ref. 16). These findings provide crucial insights into BA-mediated reproductive dysfunction. Recent metabolomic studies offer more direct evidence. Observations in porcine atretic follicles showed: (1) significantly increased BA levels; (2) abnormal primary BA biosynthesis pathways and (3) typical apoptotic features in granulosa cells (up-regulated BAX/CASPASE3 and down-regulated BCL2) (Ref. 17). Complementary studies in buffalo follicles revealed 13 differentially expressed BA sub-species between healthy and atretic follicles, particularly demonstrating accumulation of blood-derived conjugated BAs (primarily GDCA) in atretic follicles alongside decreased unconjugated BAs. Functional experiments confirmed that GDCA directly promotes granulosa cell apoptosis and inhibits steroid hormone secretion (Ref. 18). Integrating multi-species evidence, we have established a conserved BA-mediated follicular atresia mechanism: Initially, abnormal accumulation of cytotoxic conjugated BAs (especially GDCA) occurs, followed by disruption of the unconjugated-to-conjugated BA ratio, ultimately triggering apoptotic cascades that drive follicular degeneration. These discoveries not only elucidate novel mechanisms of ovarian dysfunction, but also position BA metabolism as a promising diagnostic marker and therapeutic target in reproductive aging.

## Follicular fluid BA metabolism impacts outcomes in human-assisted reproduction

A North American clinical study demonstrated that BA biosynthesis in follicular fluid plays a pivotal mediating role in connecting pro-fertility diets with assisted reproductive outcomes. Metabolomic analyses revealed this pathway is not only significantly associated with clinical pregnancy, but also functions as a central metabolic hub linking dietary patterns to reproductive outcomes: on one hand, BA metabolism significantly improves with increased adherence to fertility-enhancing diets; on the other hand, its metabolites may directly influence granulosa cell function and oocyte quality by modulating the follicular microenvironment. These findings provide the first clinical evidence supporting the physiological significance of the ‘BA–follicle metabolic axis’ in female fertility regulation, suggesting that maintaining BA metabolic homeostasis may represent a novel therapeutic target for improving female reproductive function (Ref. 19). A multi-centre cohort study investigating the efficacy of modified natural cycle IVF (MNC-IVF) revealed that BA concentrations in human follicular fluid are twice as high as those in serum, with UDCA derivatives significantly correlated with high-quality embryo development. Analysis of 303 MNC-IVF patients demonstrated that follicles yielding 8-cell embryos contained higher UDCA levels, while chenodeoxycholic acid derivatives showed positive association with embryonic fragmentation rates. These findings indicate that BA subspecies in follicular fluid directly influence oocyte quality and embryonic developmental potential by modulating granulosa cell function, particularly suggesting UDCA derivatives as novel biomarkers for predicting embryo quality and improving female fertility. This discovery provides the first clinical evidence supporting the pivotal role of the ‘BA–follicular metabolic axis’ in reproductive regulation (Ref. 20).

## BA metabolism in the diagnosis and prognosis of ovarian dysfunction: from precocious puberty to assisted reproductive outcomes

A clinical study conducted by a children’s hospital in China analysed serum BA profiles from 431 girls (241 in the training cohort and 190 in the validation cohort) using ultra-performance liquid chromatography tandem mass spectrometry. The study identified significant differences in levels of 18 BAs between girls with central precocious puberty (CPP) and normal controls, with 14 of these showing significant correlation with sex hormone levels. The researchers developed diagnostic models based on specific BA combinations: In normal-weight girls, the combination of chenodeoxycholic acid, tauro-α-muricholic acid and 6-ketolithocholic acid achieved an area under the ROC curve (AUC) of 0.885. For overweight/obese girls, hyocholic acid and taurohyocholic acid combined with basal luteinizing hormone/follicle-stimulating hormone improved the AUC to 0.914, with diagnostic accuracy reaching 75.34% and 84.09%, respectively, in the validation cohort. These findings demonstrate that serum BA profiles not only reflect sex hormone metabolic characteristics in CPP patients but also serve as novel biomarkers, potentially offering a non-invasive diagnostic alternative to the traditional reliance on invasive GnRH stimulation tests (Ref. 21).

A clinical metabolomics study comparing BA profiles between patients with diminished ovarian reserve (DOR) and controls revealed significantly lower levels of multiple BA metabolites – including lithocholic acid, chenodeoxycholic acid and UDCA – in the follicular fluid (FF) of DOR patients compared to those with normal ovarian reserve (NOR). Total BAs along with primary, secondary and unconjugated BA fractions were consistently reduced in DOR patients. Notably, these BA levels showed strong correlations with ovarian reserve parameters. A random forest diagnostic model incorporating five key BAs achieved an impressive AUC of 0.964, demonstrating the potential of BA profiling as novel biomarkers for ovarian function assessment. Complementary transcriptomic analysis of granulosa cells identified differential gene expression patterns enriched in fatty acid metabolism and ovarian steroidogenesis pathways, supporting the hypothesis that BA dysregulation contributes to DOR pathogenesis through disruption of lipid–sterol metabolism. These findings collectively highlight BA metabolism as both a diagnostic indicator and potential therapeutic target for non-invasive early intervention in ovarian dysfunction (Ref. 22).

A clinical study on PCOS sub-types revealed significant alterations in the primary BA biosynthesis pathway (impact value = 0.03267) within the follicular fluid of PCOS patients, suggesting potential involvement of BA metabolism dysregulation in PCOS pathology. Although the study did not directly evaluate the predictive value of BAs for live birth rate, combined analysis with four identified metabolic biomarkers (epitestosterone sulphate, falcarindione, lucidone C and notoginsenoside I; combined prediction AUC = 0.779) and their association with BA metabolic pathways implies that BAs may indirectly affect reproductive outcomes in PCOS women by disrupting the follicular microenvironment (including lipid metabolism and steroidogenesis). These findings provide preliminary evidence supporting the potential role of BAs as auxiliary biomarkers for live birth prediction in PCOS (Ref. 23).

## BA metabolism and PCOS

Emerging evidence highlights BA dysregulation as a pivotal mediator in PCOS, connecting metabolic dysfunction with hyperandrogenaemia and ovarian impairment through multifaceted mechanisms. This deregulation occurs both systemically in serum and locally within the follicular microenvironment, driving PCOS pathogenesis through interrelated pathways involving ER stress activation, hormonal imbalance and metabolic disturbances.

At the systemic level, serum BA profiling has emerged as a powerful tool for PCOS diagnosis and sub-group stratification. Obese women with PCOS exhibit elevated levels of TCA and taurodeoxycholic acid (TDCA), which correlate with increased fibroblast growth factor 21 (FGF-21) – a key metabolic regulator intimately linked to BA metabolism (Ref. 24). Comprehensive UPLC-MS/MS analyses confirm that PCOS patients display significantly higher glycine- and taurine-conjugated primary BAs, strongly associating with hyperandrogenaemia markers (total testosterone androstenedione) (Ref. 25). Large-scale LC/MS profiling of 408 PCOS patients reveals distinct BA alterations characterized by elevated primary and unconjugated secondary BAs, with deoxycholic acid (DCA) specifically linked to insulin resistance markers (fasting/postprandial insulin) (Ref. 26). Notably, the chenodeoxycholic acid (CDCA)–lithocholic acid (LCA)–testosterone axis demonstrates diagnostic relevance, with lean PCOS patients exhibiting distinct BA profile perturbations characterized by elevated non-12-hydroxylated BAs and increased relative abundance of CDCA that correlate with hyperandrogenaemia and reflect down-regulated hepatic CYP8B1 activity (Ref. 27).

Within the ovarian follicular microenvironment, clinical metabolomic analyses have uncovered significant elevations in primary (*p* = 0.0207) and conjugated BAs (*p* = 0.0283) in PCOS patients’ follicular fluid (Ref. 28). Specific metabolites including GCDCA, TCA and chenodeoxycholic acid-3-β-d-glucuronide (CDCA-3Gln) show marked up-regulation, with GCDCA levels positively correlating with follicle-stimulating hormone (FSH; *r* = 0.3787, *p* = 0.0017) and luteinizing hormone (LH; *r* = 0.2670, *p* = 0.0302) and CDCA-3Gln associating with antral follicle count (AFC; *r* = 0.3247, *p* = 0.0078) (Ref. 28). These findings collectively suggest that BA disturbances contribute substantially to follicular dysfunction in PCOS.

Central to BA-mediated pathogenesis is the aberrant activation of ER stress pathways. Hyperandrogenaemia triggers ER stress in granulosa cells, promoting expression of the receptor for advanced glycation end products (RAGE) and subsequent accumulation of advanced glycation end products (AGEs) – a pathological cascade effectively suppressed by the ER stress inhibitor TUDCA (Ref. 29). Pre-clinical studies in PCOS rat models demonstrate that UDCA intervention significantly improves ovarian morphology by reducing cystic follicle formation (*p* < 0.05) and increasing antral follicle count (8.5 ± 2.9 vs. 5.4 ± 1.1; *p* = 0.001) while simultaneously decreasing circulating testosterone (4.9 ± 2.8 vs. 8.8 ± 2.9 ng/mL; *p* = 0.004) and insulin levels (1.7 ± 0.08 vs. 2.1 ± 0.5 μIU/mL; *p* = 0.02) (Ref. 30). Building on these findings, a subsequent study investigated the therapeutic effects of eight BAs on a PCOS rat model, revealing that five – including TDCA and GUDCA (first reported for PCOS treatment) – not only recapitulated UDCA’s benefits, but also significantly improved additional morphological indices, corrected hormonal imbalances (e.g., P4, E2) and ameliorated ovarian histopathological changes (Ref. 31). Mechanistically, targeted proteomics identified chemerin isoforms (e.g., chemerin-157S, negatively correlated with hormone levels and follicular development) as key mediators of BA action, while network pharmacology analysis further implicated FXR binding and chemerin-associated pathways in the therapeutic effects, with BAs containing a 7β-hydroxy group demonstrating particularly enhanced efficacy (Ref. 31). Complementary metabolomic studies have reinforced the fundamental role of BA dysregulation in disrupting endocrine homeostasis, particularly through modulating androgen interactions, and directly impairing ovarian microenvironments in PCOS (Ref. 32). Collectively, these pre-clinical findings establish that specific BAs, particularly through modulation of chemerin levels and FXR signalling, represent a promising therapeutic strategy that can simultaneously address both the reproductive and metabolic abnormalities characteristic of PCOS.

These findings position BA pathways as promising diagnostic markers and therapeutic targets bridging metabolic and reproductive pathology in PCOS. The distinct regulatory roles of BA sub-classes – from the CDCA–LCA–testosterone axis to GCDCA–FSH/LH associations – underscore their clinical significance in PCOS diagnosis, mechanistic understanding and development of targeted interventions. Ongoing research continues to explore the translational potential of BA-mediated mechanisms for precision medicine approaches in PCOS management.

## The gut microbiota–BA–ovary axis: mechanistic insights into PCOS pathogenesis

**Figure 2.**
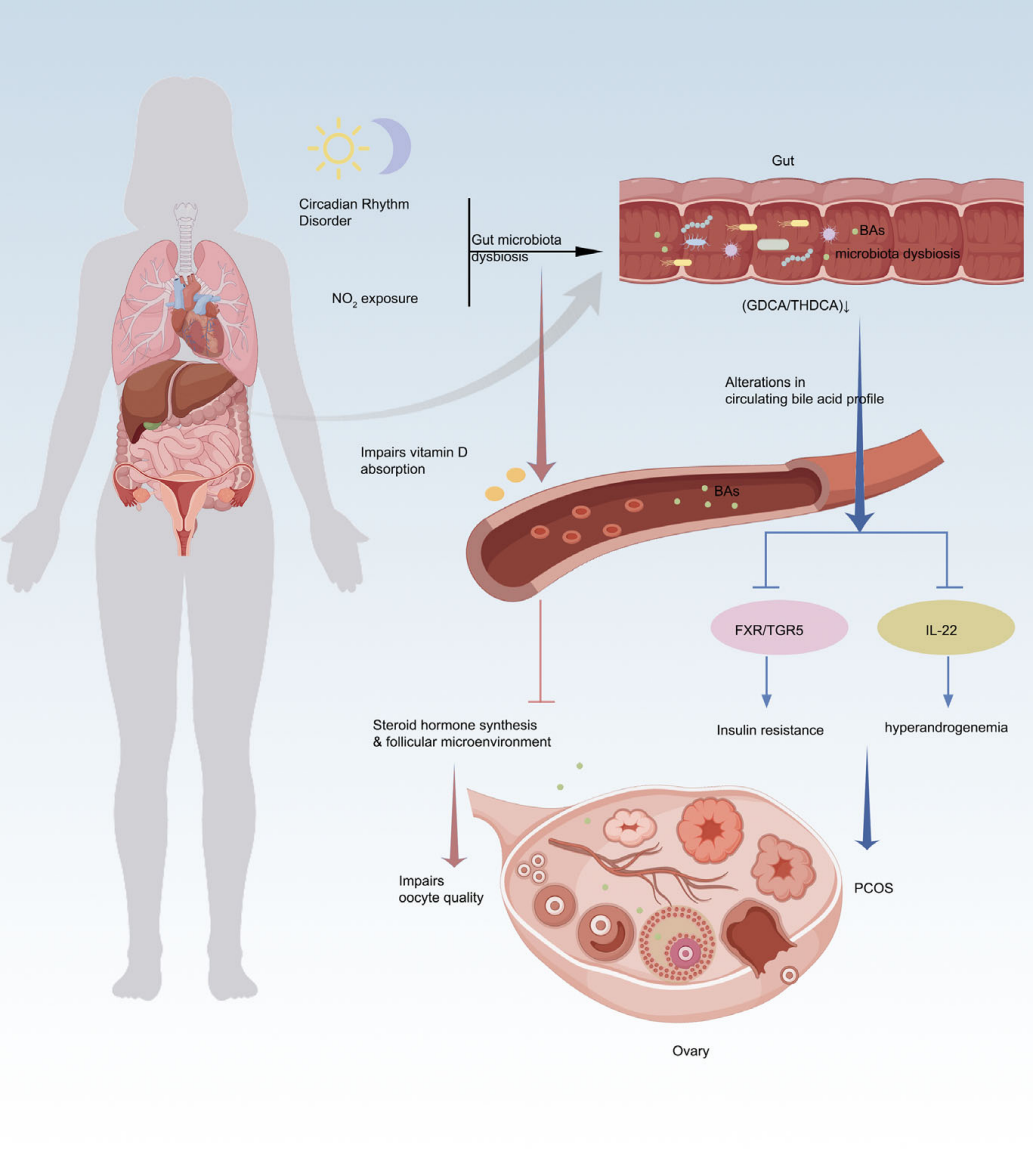
The gut–BA–ovary axis: bridging environmental stress, PCOS pathogenesis and oocyte dysfunction. **A.** Alterations in gut microbiota (e.g., reduced *Lactobacillus* and increased *Bacteroides vulgatus*) impair bile acid (BA) biotransformation, decreasing protective secondary BAs (e.g., GDCA, THDCA). BA deficiency disrupts FXR/TGR5 signalling, exacerbating insulin resistance and ovarian dysfunction, thereby promoting polycystic ovary syndrome (PCOS) manifestations. **B.** Gut microbiota dysbiosis disturbs BA metabolism; reduced secondary BAs (e.g., GDCA) suppress IL-22 secretion, triggering hyperandrogenaemia and worsening PCOS phenotypes. **C.** Circadian disruption interferes with the ‘gut–BA–vitamin D–ovary’ axis. Chronic light exposure induces gut dysbiosis, lowers lithocholic acid (LCA) levels and inhibits vitamin D absorption, impairing follicular microenvironments and reducing oocyte developmental competence. **D.** NO₂ exposure alters follicular fluid BA profiles, interacting with vitamin D₃ metabolism and steroid synthesis pathways, ultimately disrupting steroid hormone production and ovarian function.

Emerging evidence highlights the pivotal role of the gut microbiota–BAs–ovary axis in PCOS pathophysiology, where microbial-derived BAs serve as key signalling molecules that orchestrate metabolic and reproductive dysfunction. In a letrozole-induced PCOS mouse model, aromatase inhibition led to elevated androgen levels alongside significantly reduced BA concentrations, suggesting that BA dysregulation is closely associated with ovarian dysfunction (e.g., anovulation and cyst formation) and metabolic disturbances in PCOS (Ref. 33). Multi-omics and experimental studies reveal that gut dysbiosis (e.g., *Bacteroides vulgatus* overgrowth or *Lactobacillus* depletion) disrupts BA biotransformation, leading to deficiencies in protective secondary BAs like glycodeoxycholic acid (GDCA) and taurohyodeoxycholic acid (THDCA) (Refs 34–36). These BA perturbations impair FXR/TGR5 signalling, compromising glucose homeostasis via FGF15/19 and exacerbating insulin resistance – a hallmark of PCOS (Ref. 37). Notably, although NMN supplementation partially mitigated hyperandrogenism and ovarian pathology in this model, it failed to fully normalize BA metabolism, indicating that restoring BA homeostasis may be critical beyond mere hormonal modulation – its alterations likely contribute to PCOS-associated metabolic imbalance (e.g., dyslipidaemia) via the gut–liver–ovary axis (Ref. 33). Concurrently, diminished GDCA-IL-22 crosstalk (via GATA3-dependent ILC3 activation) (Ref. 34) and aberrant steroidogenesis directly disrupt ovarian folliculogenesis, linking microbial BA metabolism to hyperandrogenism and ovulatory dysfunction (Ref. 35). Interventions such as inulin-enriched synbiotics or troxerutin reprogram BA pools by expanding *Lactobacillus*/*Akkermansia*, which in turn rescue ovarian function through IL-22R1/JAK/STAT3 signalling and mitochondrial protection (Refs 36, 38). Notably, longitudinal studies position BAs as master regulators of the gut–ovary axis, where BA flux alterations precede microbial shifts during PCOS progression (Ref. 39), while maternal BA dysregulation (e.g., norDCA/DCA overflow) mediates intergenerational ovarian toxicity through carnitine depletion and follicular apoptosis. These findings collectively underscore BAs as theranostic nodes – integrating gut microbial ecology with systemic metabolic and ovarian dysfunction. Targeting BA metabolism offers a dual-pronged strategy to concurrently ameliorate insulin resistance and restore ovarian cyclicity (Refs 36, 37), bridging the gap between PCOS’s metabolic and reproductive pathologies. The gut–BAs–ovary axis thus represents a transformative paradigm for understanding and treating PCOS, emphasizing the need for precision modulation of microbial BA networks to break the vicious cycle of dysbiosis, hormonal imbalance and ovarian impairment (Refs 39, 40) (Figure 2).

## The BA–vitamin D–ovarian function axis: core regulatory mechanism underlying environmental stress-induced oocyte quality decline

An animal study elucidating the mechanisms underlying circadian disruption-induced oocyte quality decline demonstrated that sustained light exposure altered murine gut microbiota (e.g., increased *Turicibacter* abundance), leading to significant reductions in BA levels (particularly lithocholic acid, LCA). These changes impaired vitamin D absorption while decreasing serum anti-Müllerian hormone (AMH) and follicular melatonin concentrations, ultimately compromising oocyte quality and early embryonic developmental competence. Notably, exogenous administration of vitamin D3, melatonin or specific BAs (30 mg/kg LCA and 15 mg/kg NorDCA) effectively restored ovarian function by normalizing BA metabolism and attenuating intestinal oxidative stress metabolites. Notably, the vitamin D receptor (VDR) functions as a nuclear receptor for both 1,25-dihydroxyvitamin D3 and LCA and in vivo studies using VDR-knockout mice have demonstrated that VDR deletion significantly alters BAs composition and LCA distribution, particularly under conditions of BAs challenge. These findings provide direct in vivo evidence for functional crosstalk between LCA and vitamin D signalling, though the specific impact of LCA–VDR interaction on intestinal vitamin D absorption efficiency was not directly assessed in these models (Ref. 41). The study conclusively establishes BA metabolism as the pivotal modulator maintaining the integrated ‘gut microbiota–vitamin D absorption–gonadal hormone secretion–follicular microenvironment’ regulatory axis (Ref. 42). A clinical study involving 125 women undergoing in vitro fertilization at an academic fertility centre in Massachusetts, USA, revealed through metabolomic analysis of follicular fluid that nitrogen dioxide (NO_2_) exposure was significantly associated with decreased mature oocyte yields. Notably, the BA biosynthesis pathway (consistently identified in both C18 and HILIC chromatographic analyses) emerged as a key intersecting pathway. The study demonstrated that air pollutants, particularly NO_2_, may impair oocyte quality by disrupting core metabolic pathways such as BA metabolism, which concurrently participates in vitamin D3 metabolism and steroid hormone synthesis. These findings suggest that BA metabolism could serve as a potential biomarker linking NO_2_ exposure to reproductive dysfunction. The underlying mechanisms may involve: (1) modulating oxidative stress levels within the follicular microenvironment and (2) influencing follicular development through crosstalk with vitamin D metabolism and sex hormone synthesis (Ref. 43) (Figure 2).

## Therapeutic potential of BAs in ovarian function regulation and pathological interventions

### Coordinated regulation of luteal function by BAs through hormonal and cellular stress pathways

BAs (especially CDCA and TUDCA) exert comprehensive regulation on ovarian luteal function through distinct yet complementary mechanisms. Diet-derived CDCA coordinates hormonal homeostasis via the TGR5–progesterone axis: on one hand, it TGR5-dependently activates the StAR/CYP11A1 pathway to enhance ovarian steroidogenesis; on the other hand, it Stat5b-dependently inhibits the progesterone-catabolizing enzyme 20α-HSD to maintain uterine progesterone levels, thereby optimizing embryo implantation (Ref. 44). Concurrently, TUDCA demonstrates unique luteoprotective effects by modulating ER stress: in Prdx1-deficient models, it independently blocks Tm-induced unfolded protein response (↓GRP78/CHOP/caspase-3) while synergizing with antioxidants to neutralize oxidative stress and sustain progesterone secretion (Ref. 45). These two BAs constitute a dual-defence system: CDCA maintains progesterone bioavailability through endocrine signalling to enhance implantation via progesterone optimization, whereas TUDCA ensures luteal survival by mitigating ER stress-associated apoptosis, potentially preventing stress-induced luteal insufficiency in infertility. They cooperatively regulate the critical ovarian physiological pathway of ‘oxidative stress–ER stress–steroidogenesis’, providing innovative pharmacological strategies for improving reproductive outcomes.

### Modulation of folliculogenesis and oocyte maturation by BAs

Recent studies have demonstrated that chenodeoxycholic acid (CDCA), a free BA in follicular fluid, can reverse bisphenol A (BPA)-induced inhibition of oocyte maturation by activating EGFR-ERK1/2 signalling in cumulus cells, thereby ameliorating meiotic arrest (Ref. 46). Research demonstrates that UDCA (150 mg/kg) exerts dual regulatory effects in ameliorating follicular developmental abnormalities: it significantly reduces cystic/atretic follicles while markedly increasing antral follicle counts. Concurrently, this intervention effectively lowers insulin and total testosterone levels, confirming its ability to rectify follicular developmental impairment in PCOS ovaries and alleviate endocrine disturbances such as hyperandrogenaemia and insulin resistance. However, the underlying mechanisms remain to be further investigated (Ref. 30). Another study investigated the effects of TUDCA on embryonic development after fertilization in cumulus-free in vitro matured (IVM) mouse oocytes. The results showed that while TUDCA supplementation during IVM reduced oocyte maturation rates (MII rate) and pronuclear formation rates (PN rate), its addition during in vitro culture (IVC) significantly improved blastocyst formation rates in a dose-dependent manner. The researchers proposed that TUDCA likely enhances the developmental potential of IVM–IVF embryos, particularly blastocyst formation and offspring production rates, by alleviating ER stress (Ref. 47). Another study investigated the effects of TUDCA, an ER stress inhibitor, on the in vitro growth (IVG) of bovine oocytes. The results showed that while oocyte diameters remained unchanged, the antrum formation rate showed an increasing trend in the 100 μM TUDCA treatment group. Crucially, this group exhibited significant down-regulation of ER stress-related genes (*PERK*, *ATF6*, etc.) in OGCs, as well as reduced ROS levels and increased GSH content, leading to marked improvement in the maturation rate of in vitro-grown oocytes. These findings demonstrate that ER stress impairs both IVG processes and subsequent maturation capacity in bovine oocytes, while TUDCA effectively mitigates these adverse effects by modulating the ER stress pathway.

### Multidimensional protective mechanisms of BAs in ovarian pathologies

Emerging evidence highlights BAs, particularly UDCA and TUDCA, as potent modulators of ER stress-associated ovarian dysfunction across diverse pathological contexts. In ischemia/reperfusion injury, UDCA exerts antioxidant and anti-inflammatory effects, significantly reducing oxidative markers such as malondialdehyde (MDA) and preserving follicular architecture in rat ovaries post-torsion (Ref. 48). For diabetes-induced ovarian damage, TUDCA complements insulin therapy by targeting ER stress–apoptosis crosstalk – suppressing pro-apoptotic signals (ATF4/pJNK/caspases) while enhancing pro-survival XBP1 splicing, thereby mitigating follicular atresia and oxidative stress, including decreased NOX1 and MDA levels, in diabetic mice (Ref. 49). In endometriosis, TUDCA disrupts the pathogenic ‘oxidative stress–ER stress–apoptosis’ cycle in granulosa cells by inhibiting IRE1/PERK–caspase-8/3 activation, offering a therapeutic strategy to preserve follicular health (Ref. 50). Notably, in ovarian hyperstimulation syndrome (OHSS), TUDCA effectively blocks hCG-induced pathological angiogenesis by inhibiting XBP1 splicing-dependent VEGFA overproduction, as clinically validated in OHSS rat models (Ref. 51). Beyond acute pathologies, TUDCA demonstrates anti-aging potential in ovarian surface epithelium (OSE) by structurally restoring ER morphology and functionally delaying senescence via unfolded protein response (UPR) modulation, suggesting utility in menopausal ovarian maintenance (Ref. 52). Collectively, these findings underscore BAs’ triple mechanistic actions: (1) direct ER stress mitigation through UPR pathway regulation (XBP1/IRE1/PERK); (2) secondary cytoprotection via antioxidant and anti-apoptotic effects and (3) unique intervention in pathological angiogenesis (e.g., the XBP1-VEGFA axis in OHSS). Clinically, repositioning clinically approved BA derivatives like TUDCA could address unmet needs in fertility preservation (surgical/diabetic ovarian injury), assisted reproductive complications (OHSS) and age-related ovarian decline.

## Conclusions and future perspectives

In recent years, the role of BAs in ovarian physiological and pathological processes has gained increasing attention. The understanding of BA sources in the ovary has evolved from initial hypotheses of local ovarian synthesis to the current consensus that follicular fluid BAs predominantly originate from systemic circulation, where they are actively transported into the follicular microenvironment via specific transporters such as NTCP, ASBT and ABCC3. This transporter-mediated uptake mechanism plays a pivotal role in regulating ovarian function.

Pathological disruption of BA homeostasis profoundly affects ovarian physiological function. During follicular atresia, abnormal accumulation of toxic BAs including GCDCA accelerates granulosa cell apoptosis and follicular degeneration by up-regulating pro-apoptotic factors (BAX/CASPASE3) while down-regulating the anti-apoptotic protein BCL2. Follicular fluid from PCOS patients exhibits characteristic BA disorder, manifested by elevated levels of glycocholic acid (GCDCA) and TCA, and this metabolic alteration has a causal relationship with abnormally activated ER stress. BAs regulate ovarian function through diverse molecular mechanisms. They mediate direct genomic effects via nuclear receptors (FXR and TGR5) and the membrane receptor GPBAR1, while also indirectly modulating ovarian function through regulation of ER stress, redox homeostasis and vitamin D metabolism. This is exemplified by UDCA, which improves ovarian morphology and corrects endocrine abnormalities in PCOS through ER stress suppression. Furthermore, BA metabolism demonstrates regulatory effects on the ovarian microenvironment. Beyond directly influencing granulosa cell function, BAs participate in angiogenesis regulation, as demonstrated by TUDCA’s ability to counteract the aberrant VEGFA signalling pathway in ovarian hyperstimulation syndrome (OHSS), thereby suppressing pathological angiogenesis while alleviating oxidative stress. These findings collectively establish BAs as crucial modulators of ovarian physiology. They coordinate follicular development through synergistic regulation of granulosa cell viability, steroidogenesis and oocyte quality, demonstrating their systemic influence on reproductive function.

In assisted reproductive technology, the composition of follicular fluid BAs, including UDCA derivatives, has shown significant correlation with embryo quality, suggesting their potential utility as biomarkers for assessing oocyte developmental competence. Clinical studies have demonstrated that serum or follicular fluid BA profiles may serve as valuable diagnostic tools for various ovarian dysfunctions, ranging from precocious puberty to diminished ovarian reserve. Environmental factors including air pollutant NO_2_ and exogenous chemicals like bisphenol A have been shown to impair oocyte quality through disruption of BA metabolic pathways, whereas specific dietary patterns such as fertility-promoting diets can optimize intra-follicular BA profiles to improve reproductive outcomes. Emerging evidence indicates that gut dysbiosis-induced alterations in BA metabolism, such as reduced GDCA/THDCA levels, can disrupt FXR/TGR5 signalling and exacerbate PCOS-associated hyperandrogenism and insulin resistance, thereby establishing the gut–BAs–ovary axis as a promising novel therapeutic target. Therapeutic interventions targeting this axis, including synbiotics or BA-based therapies, have shown promise in concurrently addressing both metabolic and reproductive abnormalities in PCOS. These provide a solid theoretical foundation for the clinical application of BAs.

Despite significant progress, critical questions remained unresolved. Future research should focus on elucidating the precise mechanisms of BA dynamic transport in the ovary, including how transporters such as FABP6 and ASBT were hormonally regulated and whether they exhibited stage-specific expression patterns during folliculogenesis. Secondly, deciphering the interconversion and regulatory mechanisms among BA subtypes in follicular fluid to inform drug development. Thirdly, Optimization of clinical translation requires standardization of BA detection protocols, development of personalized therapeutic regimens (including precision modulation of the gut–BAs–ovary axis via microbiome transplantation or FXR/TGR5-targeted agents), and determination of optimal clinical indications and intervention timing for UDCA/TUDCA administration in assisted reproductive technologies.

In conclusion, BAs have transcended their traditional role as digestive adjuncts to emerge as key regulators of ovarian function modulation. Future research should focus on elucidating their underlying mechanisms and facilitating clinical translation, which would not only provide innovative diagnostic and therapeutic approaches for various reproductive disorders including PCOS, precocious puberty and premature ovarian failure, but also potentially enable development of targeted therapeutic agents based on BA metabolism to achieve precise regulation throughout a woman’s reproductive lifespan – from pubertal development through perimenopausal functional maintenance.
